# Bone and Infections: An Osteoimmunological Interplay

**DOI:** 10.3390/ijms27062602

**Published:** 2026-03-12

**Authors:** Emanuela Galliera, Luca Massaccesi, Nicola Logoluso, Laura Mangiavini, Giuseppe Peretti, Massimiliano Marco Corsi Romanelli

**Affiliations:** 1 Department of Biomedical Sciences for Health, Università Degli Studi di Milano, 20122 Milan, Italy; luca.massaccesi@unimi.it (L.M.); laura.mangiavini@unimi.it (L.M.); giuseppe.peretti@unimi.it (G.P.); mmcorsi@unimi.it (M.M.C.R.); 2 IRCCS Galeazzi Orthopedic Institute, 20157 Milan, Italy; nicola.logoluso@gmail.com; 3 Department of Clinical and Experimental Pathology, IRCCS Istituto Auxologico Italiano, 20095 Milan, Italy

**Keywords:** osteoimmunological biomarkers, infection, bone remodeling

## Abstract

Osteoimmunology examines the bidirectional interactions between the skeletal and immune systems, focusing on the mechanisms by which immune cells regulate bone homeostasis and how the bone microenvironment modulates immune responses. Chronic inflammation is a major driver of bone loss, and infections of bacterial or viral origin perturb bone remodeling with consequences for host defense. In infected bone tissue, immune cell infiltration and the release of cytokines and soluble mediators alter the activity of osteoclasts and osteoblasts, thereby promoting bone erosion and structural remodeling. Recent studies highlight how immune dysregulation contributes to the pathogenesis of osteomyelitis and other infection-associated bone disorders, implicating specific inflammatory pathways and cellular interactions as potential therapeutic targets. This review synthesizes current evidence on direct and indirect mechanisms by which infection affects bone, identifies gaps in mechanistic understanding, and discusses implications for diagnosis and intervention.

## 1. Introduction

The immune system plays a crucial role in bone metabolism through complex interactions between immune cells and bone cells. This relationship is central to the field of osteoimmunology, which explores how immune signaling affects bone formation and resorption. Osteoimmunology is an emerging field that explores the intricate relationship between the skeletal system and the immune system, investigating how immune cells influence bone health and disease, and vice versa. This interdisciplinary science has led to breakthroughs in understanding conditions like osteoporosis, rheumatoid arthritis, bone metastases, and recently bone infections [1,2]. Osteoimmunology plays a crucial role in understanding bone infections. When an infection occurs in bone tissue, immune cells infiltrate the affected area, releasing cytokines and signaling molecules that influence osteoclast and osteoblast activity. This immune response can lead to bone erosion and remodeling, and recent research highlights how immune dysregulation contributes to bone infections and related diseases [3,4,5]. The present review summarizes the latest updates about the osteoimmunological aspects of infection affecting bone directly and indirectly. The effect of bacterial and viral infections on bone formation and resorption is briefly summarized in Table 1 and is further described in the following sections of this review.

## 2. Bone Infections

Bone cells can be regulated by a variety of immune factors, including immune regulators. Bone infections are very complex diseases, where both local bone cells and the infecting pathogen can modify the immune response [6]. In infections, these factors are dysregulated and can have effects on bone metabolism and remodeling. Infections can affect bone directly through bone tissue infection or indirectly via the effects of the inflammatory response to infection on bone [7].

Since chronic inflammation is associated with bone loss, the alteration of bone homeostasis by bacterial or viral infection influences host defense during the course of infectious disease. Bone tissue infection, also known as osteomyelitis, occurs when bacteria or fungi invade the bone, leading to inflammation and damage. Most osteomyelitis patients are infected with *Staphylococcus aureus*, which colonizes bone tissue via the bloodstream, by direct inoculation following trauma or surgery or subsequent spread of a contiguous infection [8,9].

Periprosthetic joint infections (PJIs) are serious complications that can occur after joint replacement surgery, affecting the artificial joint and surrounding tissues [10]. These infections can be acute (developing within weeks of surgery) or chronic (emerging months or years later). The increasing numbers of joint replacement have resulted in an increase in PJI frequency. PJI affects more than 2% of arthroplasty patients, and its prevalence continues to rise due to the growing number of joint replacement surgeries [11,12]. Along with joint prosthetics, other types of bone implant are used in surgery to fix fractures. This implant can be susceptible to infections, leading to implant-associated osteomyelitis [13,14] (IAO). When an implant-related infection is established, it is unlikely that it can be resolved with antibiotic treatments, often requiring revision surgery [15].

Periodontitis is an oral inflammatory disease that occurs when plaque and tartar buildup cause inflammation and infection in the gums [13]. Innate immune cells stimulated by oral bacteria produce a variety of inflammatory cytokines that contribute to stimulating osteoclastogenesis, leading to bone loss [16]. It is characterized by periodontal pocket formation resorption [17]. Over time, this can lead to the destruction of the bone supporting the teeth, resulting in loose teeth or even tooth loss.

## 3. Osteoimmunolgical Mediators in Bone Infections

### 3.1. RANKL-RANK-OPG System

The RANKL–RANK–OPG system plays a crucial role in bone metabolism, immunity, and even bone infections [18]. This system is essential for bone homeostasis, as well as immune function. RANKL (Receptor Activator of Nuclear Factor Kappa-B Ligand) is a protein that stimulates osteoclast formation, leading to bone resorption. RANK (Receptor Activator of NF-κB) is the receptor for RANKL, found on osteoclast precursors and immune cells. RANKL binds to its receptor RANK on osteoclast precursors, stimulating their maturation into active osteoclasts that break down bone. OPG (Osteoprotegerin) is a decoy receptor that binds to RANKL, preventing it from activating RANK and inhibiting bone resorption. OPG acts as a decoy receptor, binding to RANKL so that it cannot activate RANK, effectively reducing osteoclast activity. This helps prevent excessive bone resorption, ensuring a healthy balance between bone loss and formation [19,20,21,22,23].

The RANKL–RANK–OPG system plays a significant role in bone infections, particularly in osteomyelitis [24]. Chronic infections, especially bacterial osteomyelitis, trigger persistent inflammation and increase RANKL activity, leading to bone degradation. During infections, inflammatory cytokines (like TNF-α and IL-6) stimulate RANKL production, leading to excessive osteoclast activation and bone resorption. In some cases, infection can lead to OPG downregulation: OPG levels decrease, reducing its protective effect against RANKL and allowing uncontrolled bone destruction. Bacterial pathogens like *S. aureus* can stimulate osteoclastogenesis by inducing RANKL and reducing OPG expression, as confirmed by recent evidence showing that RANKL-mediated osteoclast formation is required for bone loss in a murine model of *S. aureus*-induced osteomyelitis [25]. The RANKL/OPG ratio is a key determinant of the degree of bone resorption through osteoclast activity. Vertebral osteomyelitis has been recently described as being characterized by an increased RANKL/OPG ratio, both at the expression and protein levels [25].

The RANKL–RANK–OPG system is linked to immune responses, influencing how macrophages and dendritic cells react to infections. RANKL enhances the survival and function of dendritic cells (DCs), which are essential for antigen presentation and T-cell activation [26,27]. RANKL signaling is also critical for lymphoid organ formation, including lymph nodes and Peyer’s patches, which are key sites for immune responses. RANKL influences self-tolerance by modulating regulatory T cells, helping prevent autoimmune reactions [28,29,30].

### 3.2. Cytokines and Chemokines

Bone infections trigger a complex immune response, where cytokines play a crucial role in bone destruction and repair. Proinflammatory cytokines such as TNF-α, IL-1β, and IL-6 drive osteoclast activation, leading to bone resorption. On the other hand, anti-inflammatory cytokines including IL-10 and TGF-β help regulate inflammation and promote bone healing [31,32]. IL-6 is rapidly produced in response to bacterial infections, triggering immune cell activation and promoting inflammation. IL-6 induces both osteoclast activation and osteoblast dysfunction: IL-6 stimulates RANKL expression, leading to osteoclast differentiation and bone resorption. Chronic IL-6 exposure can impair osteoblast function, reduce bone formation, and contribute to bone loss. IL-6 is also involved in immune modulation: IL-6 helps recruit macrophages and neutrophils to infection sites, helping in pathogen clearance but also contributing to exacerbating bone destruction [33,34,35].

Emerging evidence shows that osteoblasts and osteoclasts actively participate in the immune response by secreting a variety of chemokines [36,37]. For instance, in staphylococcal osteomyelitis, the osteoblast secretome includes a range of molecules that help summon neutrophils, monocytes, and lymphocytes to the site of infection [25]. Research has demonstrated that the pattern and timing of chemokine release can differ depending on the pathogen involved. *Staphylococcus aureus* can stimulate a profile of secreted chemokines different from that of other infections, such as tuberculous osteomyelitis, suggesting pathogen-specific immune strategies in bone tissue [38].

#### 3.2.1. CCL2/CCR2 Axis

CCL2 (Monocyte Chemoattractant Protein-1, MCP-1) is one of the most well-studied chemokines in the context of bone inflammation. By binding to its receptor CCR2, it recruits monocytes and macrophages to the infection site. In osteomyelitis—often caused by pathogens such as *Staphylococcus aureus*—this pathway helps bring in cells crucial for bacterial clearance. However, an unchecked influx of monocytes can also contribute to an excessive inflammatory response, which may disrupt the balance of bone remodeling by favoring resorption over formation [39,40,41]. This axis is therefore a double-edged sword in managing both infection and inflammation [42].

#### 3.2.2. CXCL8 (IL-8) and Its Receptors: CXCR1 and CXCR2

CXCL8 (Interleukin-8) is another central chemokine in bone infections. It signals primarily through CXCR1 and CXCR2, which are abundantly expressed on neutrophils. The activation of these receptors drives a rapid neutrophil response—a key element of the early innate immune defense during bacterial infections [43,44,45]. While the robust neutrophilic infiltration helps eliminate pathogens, it can also lead to collateral damage in bone tissue if the inflammation is not resolved properly [46].

In the setting of bacterial osteomyelitis, osteoblasts have been shown to secrete a specific subset of chemokines, including CXCL1, CXCL2, CXCL3, and CXCL5. These members primarily activate CXCR2 on neutrophils. Their production is typically induced via pathogen recognition receptors (such as Toll-like receptors) and downstream signaling cascades like NF-κB or MAPKs. The resulting chemotaxis of neutrophils is central both to effective microbial clearance and, if overactive, to inflammatory bone degradation [43].

The CXCL10/CXCR3 pathway is more often associated with chronic inflammatory states and is particularly noticeable in conditions like tuberculous osteomyelitis. CXCL10 not only recruits T cells but also helps maintain a prolonged inflammatory milieu, which can contribute to granuloma formation. This sustained immune response, while crucial in containing certain infections, may also predispose individuals to chronic bone damage if unresolved [47,48].

Other chemokine–receptor interactions, such as CCL3/CCR1 [49] and CCL7/CCR2 [50,51], also play roles in modulating the immune response during bone infections [52]. These pathways participate in recruiting diverse leukocyte populations, each contributing to the proinflammatory environment. The overall outcome involves a finely tuned network where the beneficial antimicrobial effects of immune cells are balanced against the risk of excessive inflammation and subsequent bone resorption.

#### 3.2.3. Toll-like Receptors

Many of these chemokine pathways are activated in response to pathogen-associated molecular patterns (PAMPs) recognized by Toll-like receptors on bone cells and resident immune cells [53,54]. Toll-like receptors (TLRs) are essential components of the innate immune system, acting as the first line of defense by recognizing conserved microbial structures [55,56]. In the context of bone infections, such as osteomyelitis, TLRs do more than simply detect pathogens—they shape the local immune and inflammatory responses that significantly influence bone remodeling and homeostasis. Upon ligand binding, TLRs activate intracellular signaling cascades—most notably the NF-κB and MAPK pathways. These signaling events lead to a rapid transcriptional upregulation of proinflammatory cytokines such as IL-1β, TNF-α, and IL-6. While these cytokines are critical for initiating an effective immune response to clear the infection, they also stimulate osteoclast differentiation and activation, which can disrupt the delicate balance of bone remodeling [57,58,59]. Thus, excessive or dysregulated TLR signaling may inadvertently contribute to bone resorption and structural damage in chronic infections.

TLR2 is primarily activated by components derived from Gram-positive bacteria (e.g., peptidoglycan and lipoteichoic acids), which are abundant in pathogens like *Staphylococcus aureus* that commonly cause bone infections. Activation of TLR2 on immune cells and even on bone cells (such as osteoblasts) leads to the production of proinflammatory cytokines and chemokines [58]. Notably, genetic variability in the TLR2 gene—such as the R753Q polymorphism—has been associated with increased susceptibility to osteomyelitis, suggesting that differences in TLR2 signaling can influence an individual’s risk of developing bone infections [60,61].

Although TLR4 is best known for its role in recognizing lipopolysaccharide (LPS) from Gram-negative bacteria, it also contributes to inflammatory bone resorption in diseases like periodontal disease. TLR4 activation leads to the induction of inflammatory mediators, including prostaglandin E2 (PGE2), through its downstream signaling pathways (primarily NF-κB and MAPK) [62,63,64]. The resultant increase in PGE2 and cytokine production promotes osteoclast differentiation, thereby enhancing bone resorption. While the role of TLR4 is more clearly defined in periodontal inflammation [65,66], its involvement in broader contexts of inflammatory bone loss underscores its importance in bone infection-related pathology.

TLR9 recognizes unmethylated CpG motifs common in bacterial and viral DNA. In the setting of bone infections, TLR9 has been implicated in the chronic inflammatory response [67]. Studies using mouse models of *Staphylococcus aureus* osteomyelitis have illustrated that, while deficiency in TLR2 alone might not markedly prevent bone damage, the combined deficiency of both TLR2 and TLR9 results in a significant reduction in trabecular bone loss. This suggests that TLR9 may function complementarily with TLR2 to drive osteoclastogenesis and sustain inflammatory responses, leading to bone degradation [68].

## 4. Bone Infection: Osteoimmunological Effect on Bone Cells

**Figure 1:**
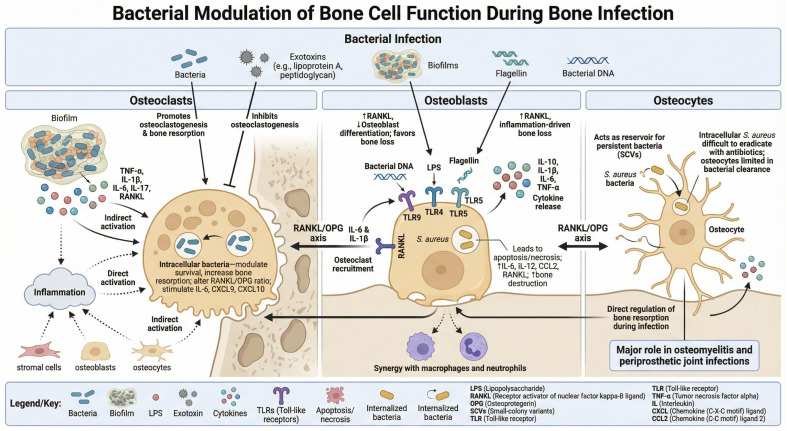
Osteoimmunological effect of bone infections on osteoclasts, osteoblasts, and osteocytes.

Bone remodeling is a dynamic process that maintains bone strength and mineral balance, involving specialized bone cells: osteoclasts, osteoblasts, and osteocytes, as described in Figure 1.

### 4.1. Osteoclasts

It has been reported that osteoclastogenesis is affected by proinflammatory cytokines and chemokines produced during the inflammatory response. Therefore, the inflammatory response against pathogens (viruses or bacteria) can affect bone resorption through osteoclast activity. During infections, osteoclast homeostasis can be disrupted by the inflammatory response induced by bacterial invasion and multiple bacterial virulence factors, including exotoxins, endotoxins such as LPS, and the formation of biofilms. Exotoxins such as Lipoprotein A and peptidoglycan promote osteoclastogenesis through different pathways [69,70]. Bacterial proteins can have opposite effects on osteoclasts, either inducing or preventing osteoclastogenesis. Bacterial DNA inhibits osteoclast formation, while endotoxin LPS enhances osteoclast formation [71]. One of the most important effects of bacterial invasion is the formation of a biofilm at the infection site. A biofilm is a bacterial structure that can attach to a non-living surface (necrotic bone for implants), which prevents the recruitment of immune cells and the arrival of antibiotics to the site of infection, thus making bone infection difficult to cure. Moreover, bacterial biofilms contain bacterial products that trigger the release of inflammatory cytokines such as TNF-alpha, IL-1 beta, IL-6, and IL-17 and RANKL in the bone microenvironment, thus indirectly promoting osteoclast formation [72,73]. Bacteria can affect osteoclast formation through several ways: they can directly stimulate the inflammatory response, ultimately leading to osteoclastogenesis; they indirectly affect osteoclast formation by targeting bone stromal cells to increase RANKL, controlling the RANKL/OPG ratio and the production of inflammatory factors, like TNF-alpha and IL-6, in the bone microenvironment by osteoblast and osteocytes [74,75,76,77]. Some bacteria can also be internalized by osteoclasts, thus directly affecting their survival and bone resorption activity. Internalized bacteria increase the RANKL/OPG ratio within the bone microenvironment, thereby indirectly promoting osteoclastogenesis or regulating the expression of inflammatory factors, such as IL-6, CXCL9, and CXCL10 [78,79].

### 4.2. Osteoblasts

Several recent reports indicated that osteoblasts could contribute to the inflammatory and immune responses to pathogen infections [80,81]. Osteoblasts express RANKL upon IL-6 and IL1 beta stimulation, as well as the chemokine CCL2, in order to recruit monocytic precursors, such as osteoclasts, thereby contributing to inflammation-induced bone resorption. In addition, osteoblasts produce inflammatory cytokines, including IL-10, Il1 beta, IL-6, and TNF-alpha, thereby acting as a direct player in the inflammatory response [81]. Osteoblasts are also able to respond directly to bacterial products. In fact, osteoblasts have been reported to express many Toll-like receptors targeting LPS (TLR4), flagellin (TLR5), and bacterial DNA (TLR9) [82,83]. LPS is a bacterial cell component with several roles in the inflammatory response. In bone tissue, LPS contributes to bone resorption in diverse ways: on the one hand, LPS induces RANKL production by osteoblasts, thereby stimulating bone resorption; on the other hand, LPS can inhibit osteoblast differentiation, thus shifting the balance of bone remodeling toward bone loss [84,85]. Flagellin is a component of Gram-positive and Gram-negative bacteria, and it is a ligand of TLR5. Recent in vitro reports indicated that, in osteoblasts, TLR5 stimulation by flagellin can induce RANKL production, playing a role as a new regulatory factor of inflammation-induced bone loss [86].

Similar to osteoclasts, osteoblasts can also internalize the pathogen. *Staphylococcus aureus*, which is one of the main pathogens in osteomyelitis and PJI, is internalized by osteoblasts. Upon internalization, *Staphylococcus aureus* induces multiple responses in osteoblasts. It induces cell death: infected osteoblasts activate apoptosis and necrosis pathways. Infected osteoblasts also produce inflammatory cytokines and chemokines, including IL-6, IL-12, CCL2, and RANKL, leading to an increase in inflammatory bone loss [87,88].

Taken together, cytokines and chemokines released by activated or infected osteoblasts can contribute to synergy with the mediators released by macrophages and neutrophils during bone infections.

### 4.3. Osteocytes

Osteocytes, the most abundant cells in bone tissue, play a significant role in bone infections, particularly in conditions like osteomyelitis and periprosthetic joint infections (PJIs) [89]. They can be involved in both the inflammatory response and the long-term persistence of bacteria within bone. While osteocytes can contribute to recruiting immune cells to clear the infection, they may also serve as a reservoir for bacteria, potentially contributing to the persistence of the infection. Research suggests that *Staphylococcus aureus* can invade and persist within osteocytes, forming small-colony variants (SCVs) that contribute to chronic and difficult-to-treat infections [90]. Unlike osteoblasts, which have strong antimicrobial responses, osteocytes struggle to clear intracellular infections, making them a reservoir for persistent bacteria. This persistence can lead to prolonged inflammation and bone destruction, complicating treatment efforts. Additionally, studies indicate that conventional antibiotics may have limited effectiveness against intracellular *S. aureus* infections in osteocytes, highlighting the need for new therapeutic approaches.

Furthermore, osteocytes can directly regulate bone resorption (osteolysis) during bone infection, which can further contribute to bone damage.

Recent evidence [91] shows that osteocytes stimulated with bacterial pathogen-associated molecular patterns (PAMPs) directly drive bone resorption through an MYD88-regulated signaling pathway, which, in turn, upregulates RANKL production. In this way, osteocytes directly regulate inflammatory osteolysis in bone infection, suggesting that MYD88 and downstream RANKL regulators in osteocytes are therapeutic targets for osteolytic osteomyelitis and periodontitis.

## 5. Effect of Bacterial Infection on Bone Remodeling: Lessons from Prosthetic Joint Infections (PJIs) and Other Bone Infections

Bone remodeling is a dynamic process involving osteoclast-mediated bone resorption and osteoblast-driven bone formation. Bacterial infections significantly alter this balance, especially in conditions like osteomyelitis or chronic apical periodontitis. Bacterial components like lipopolysaccharides (LPSs) activate immune responses that increase osteoclastogenesis. This leads to excessive bone resorption, weakening the bone structure [92].

During acute inflammation, inflammatory cytokines such as TNF-α and IL-1β released during infection inhibit osteoblast differentiation and function, reducing bone formation.

When the infection is persistent, it creates a chronic proinflammatory environment that sustains bone loss and prevents healing. This is especially problematic in chronic infections like periodontitis [93]. These effects interfere with bone regeneration: infected bone defects are difficult to repair due to microbial interference with tissue regeneration. Therefore, healing strategies must address both infection control and osteogenesis [94]. The clinical consequences can be a delay in the healing process because infections slow down recovery after fractures or surgeries.

In this way, bone loss due to chronic infections leads to irreversible bone damage. This complication represents a therapeutic challenge that requires integrated approaches—antibiotics, immune modulation, and regenerative techniques.

Treatment strategies aim to overcome this problem by combining antibacterial therapies with bone grafting or biomaterials, which can improve outcomes. Animal models reveal that bacterial burden directly correlates with impaired healing [95].

An example of osteoimmunology’s input to bone infection diagnosis and clinical management is prosthetic joint infection (PJI) [96]. PJI occurs when bacteria colonize a joint prosthesis, leading to inflammation, pain, and implant failure. PJI is one of the most feared complications of joint arthroplasty due to its complexity and impact on patient outcomes [97,98]. Bacteria form biofilms on prosthetic surfaces, evading immune detection and resisting antibiotics. Common pathogens include *Staphylococcus aureus*, *Staphylococcus epidermidis*, and other biofilm-forming bacteria. The chronic inflammation induced by PJI can impair bone remodeling and healing, complicating infection resolution [99], [one hundred]. The inflammatory response also affects bone–immune crosstalk: cytokines like IL-6, TNF-α, and RANKL influence both immune responses and osteoclast activity, affecting bone resorption and implant stability. For these reasons, PJI is considered one of the most challenging complications of joint arthroplasty, and appropriate diagnosis and management are crucial to prevent excess morbidity and restore adequate function, but there is still no “gold standard” for PJI diagnosis and prognosis [99,100]. Therefore, there is a pressing need for improved diagnostic tools.

The osteoimmunology approach to PJI diagnosis has recently defined a panel of new inflammatory markers involved in the monocyte-/macrophage-mediated inflammatory response, such as OPN (osteopontin), the chemokine CCL2, and suPAR, as well as the infection biomarker sCD14st, that could help improve PJI diagnosis and prognosis [101,102,103]. Several studies also show that gene expression patterns of TLRs are altered in PJI, suggesting their involvement in the host response [104,105,106]. Therefore, TLRs may help distinguish between aseptic loosening and infection, which is a major diagnostic challenge in orthopedics. TLR expression profiling could serve as a biomarker for PJI, improving diagnostic accuracy. For example, elevated levels of TLR2 and TLR4 have been associated with bacterial infections in joint tissues [107]. Consistently, a recent clinical study showed that PJI patients displayed high Toll-like receptor 2 (TLR2) serum levels, correlating with canonical inflammatory markers CRP, IL-6, tumor necrosis factor alpha TNF-α, and IL-1 [108]. Therefore, TLR2 serum levels could be considered a new potential diagnostic tool in the early detection of PJI.

Despite advances in therapeutic approaches, bone and joint infections remain clinically challenging owing to persistently high rates of treatment failure and recurrence. Osteomyelitis represents one of the most demanding conditions encountered by orthopedic surgeons: it is defined by infection-driven inflammation of bone and adjacent soft tissues that culminates in progressive bone destruction. Although uncommon, osteomyelitis carries substantial morbidity because conventional management frequently fails to eradicate infection, often resulting in permanent functional impairment or the need for limb amputation [109].

Diagnosis of osteomyelitis remains challenging due to the intrinsic limitations of current diagnostic modalities. Conventional radiologic techniques, including plain radiography and magnetic resonance imaging (MRI), lack definitive diagnostic specificity, and MRI utility is frequently compromised in the presence of metallic implants. Although bone culture is considered the diagnostic gold standard, reported false-negative rates approach 40%, clinical presentation is heterogeneous, and routine serum inflammatory markers exhibit limited sensitivity and specificity in the context of orthopedic infections. Consequently, there is an unmet need for circulating biomarkers that provide greater diagnostic and prognostic resolution in the specific clinical setting of osteomyelitis.

Osteoimmunological approaches previously applied to prosthetic joint infection have recently been extended to osteomyelitis. In this context, CD14ST has been evaluated alongside a panel of serum osteoimmunological markers to characterize the inflammatory milieu and its direct effects on bone remodeling. These investigations have identified a potential diagnostic role for SuPAR, CCL2, the anti-inflammatory cytokine IL-ten, and the Wnt pathway inhibitors DKK1 and sclerostin, while the RANKL/OPG ratio has shown notable diagnostic value. Moreover, CCL2 and SuPAR have demonstrated short-term prognostic utility in the immediate postoperative period [110].

Periodontitis is a chronic, immune-driven inflammatory disease where host immune responses and bone cells interact and cause net alveolar bone loss. Biomarkers that reflect inflammation, osteoclast activation, and bone formation/resorption can help with diagnosis, prognosis, monitoring treatment response, and identifying therapeutic targets. Immune cells (T cells, B cells, and macrophages) release cytokines that upregulate RANKL, a critical molecule that promotes osteoclast differentiation. Periodontal ligament cells respond to inflammatory signals by producing more RANKL, tipping the balance toward bone resorption. Osteocytes, once thought to be passive, actively contribute to disease progression by modulating bone remodeling signals. In periodontitis, dysbiosis of the oral microbiome triggers an inflammatory response dominated by cytokines such as IL-17 and TNF-α. IL-17, from Th17 cells, amplifies inflammation and strongly induces RANKL expression on immune and stromal cells. TNF-α, produced by macrophages and T cells, synergizes with RANKL to accelerate osteoclast differentiation and tissue breakdown. The central osteoimmune pathway involves the RANK/RANKL/OPG axis: RANKL binds to RANK on osteoclast precursors, driving their maturation and promoting bone resorption. OPG acts as a decoy receptor that neutralizes RANKL and protects bone [111].

In periodontitis, inflammation increases RANKL and reduces OPG, creating a high RANKL/OPG ratio that leads to excessive osteoclast activity and alveolar bone loss, the hallmark of the disease. In periodontitis, cytokines such as IL-17 and TNF-α, along with the RANK/RANKL/OPG system, serve as valuable biomarkers for assessing disease activity and severity. Elevated RANKL and a high RANKL/OPG ratio in gingival crevicular fluid or periodontal tissues indicate active osteoclastogenesis and ongoing bone loss. Increased levels of IL-17, TNF-α, and related proinflammatory mediators correlate with inflammation intensity and can help distinguish active from stable disease. Together, these markers support early detection, monitoring of disease progression, and evaluation of treatment response [112].

The effects of bacterial infection on bone remodeling are summarized in Table 2.

Understanding osteoimmunology can also improve PJI management by targeting immune pathways to enhance infection clearance without harming bone integrity, developing biomaterials that resist biofilm formation [113,114,115] and modulate immune responses, and personalizing treatment based on patient immune profiles and bone health status.

## 6. Effect of Viral Infection on Bone Remodeling: Lessons from HIV to COVID-19

Viral infections can significantly disrupt bone health by altering bone remodeling, reducing bone mineral density, and triggering inflammatory responses that impair skeletal integrity. Viruses stimulate the release of proinflammatory cytokines [116,117,118] such as IL-6 and TNF-α, which, in turn, promote osteoclastogenesis via the RANKL pathway and inhibit osteoblast differentiation and survival. This immune response can lead to bone demineralization and structural weakening, especially in chronic or severe infections.

The effect of viral infection on bone remodeling is summarized in Table 2. Since its discovery, HIV has progressively become a manageable chronic condition. This unveiled new clinical challenges associated with aging-related pathologies, including bone disease: HIV infection disrupts bone remodeling by promoting chronic inflammation, altering immune signaling, and affecting osteoblast and osteoclast activity, thus leading to an increased risk of osteoporosis and fractures [119,120,121]. HIV triggers persistent inflammation, elevating cytokines like TNF-α and IL-6, which stimulate osteoclasts and suppress osteoblasts. HIV proteins may directly interfere with bone cell function and differentiation, contributing to bone loss. HIV can also alter RANK/RANKL/OPG signaling [122,123]. Therefore, after infection, promotion of osteoclastogenesis and osteolytic activity has an effect on osteoblasts and their precursor cells, leading to exacerbated senescence of mesenchymal stem cells (MSCs). HIV infection boosts osteoclast formation and bone resorption while impairing osteoblasts and accelerating MSC senescence. Dysfunctional B and T cells reduce OPG production, and chronic inflammation increases RANKL-expressing B cells while decreasing OPG-producing ones. This shift raises the RANKL/OPG ratio and promotes bone loss [124,125,126].

The clinical consequence is a significant increase in osteopenia and osteoporosis in people living with HIV (PLWH), with fracture risk up to three times higher than in HIV-negative individuals [127,128].

More recently, COVID-19 has been linked to increased bone fragility and susceptibility to bone infections due to immune dysregulation and systemic inflammation [129,130,131,132]. COVID-19 triggers a cytokine storm, releasing high levels of inflammatory mediators like IL-6 and TNF-α, which can disrupt bone remodeling and weaken the bone structure [133].

This immune imbalance may impair the body’s ability to fight off bone infections, especially in vulnerable populations. Studies show that long-term COVID-19 infection is associated with reduced bone mineral density and increased fracture risk, and it potentially makes bones more susceptible to infection. Severe cases of COVID-19 have been linked to accelerated bone loss, particularly in older adults and those with pre-existing conditions [125]. Research highlights a change in osteoclast and osteoblast activity post-COVID-19, suggesting that the virus may directly affect bone metabolism [134]. Biomarkers like RANKL and OPG are being studied to understand the osteoinflammatory response in COVID-19 patients [135]. RANKL/OPG showed a good correlation with the bone fragility maker FGF23, suggesting a role as a dependable maker of bone fragility in COVID-19 patients, providing useful and comprehensive information about inflammation-induced bone loss. This aspect is extremely important in elderly patients undergoing orthopedic surgery, who can present more severe effects of COVID-19 and an increased level of age-induced bone fragility.

Other osteoimmunological biomarkers have drawn increasing interest, mirroring the approach taken in prosthetic joint infection (PJI) research. Among them, suPAR and sCD14st have emerged as strong indicators of PJI and as valuable diagnostic and prognostic biomarkers for COVID-19 outcomes [136]. These receptors connect immune activation with bone metabolism, particularly in conditions such as COVID-19, where inflammation can contribute to bone fragility and infection-related damage. Tracking sCD14ST together with suPAR may help reveal immune dysregulation and bone complications in inflammation-driven diseases.

## 7. Future Directions of Therapeutic Strategies

Bone infections such as osteomyelitis and implant-related infections are notoriously difficult to treat due to biofilm formation, intracellular bacterial persistence, and dysregulated inflammation. Immune modulation offers a promising adjunct to conventional therapies by targeting host responses to enhance clearance and preserve bone integrity. An overview of immune modulation strategies for preventing and treating bone infections is described in Table 3.

Recent research into chronic, implant-related bone infections has focused on immune modulation as a means of preventing the transition to a biofilm-dominated state and chronic inflammation.

Beyond direct cytokine blockade, strategies that modulate the overall immune profile—such as immune checkpoint inhibitors adapted from cancer therapy—are under investigation to reactivate effective local immune responses while reducing destructive inflammation [137].

Advances in cytokine-based diagnostic models (e.g., those that differentiate infection from cytokine release syndromes) could eventually guide targeted therapy by identifying which cytokines are most dysregulated in each patient. Such personalized approaches could help tailor therapy to the exact inflammatory profile of the bone infection [138].

Blocking proinflammatory cytokines (e.g., TNF-α, IL-1β, IL-6, and IL-17) or enhancing anti-inflammatory ones can rebalance the immune response. IL-9 has emerged as a novel target for mitigating osteoclast-driven bone loss [139].

Anti-RANKL agents (e.g., denosumab) reduce osteoclastogenesis and bone resorption. Recent studies suggest that combining anti-RANKL therapy with antibiotics may prevent joint destruction in septic arthritis models [140]. Denosumab (human monoclonal antibody to RANKL) is approved for osteoporosis, prevention of skeletal-related events in bone metastases, and other bone-loss indications; it is administered subcutaneously and has well-documented effects on BMD and fracture risk.

The mechanism of action is based on the direct neutralization of RANKL, which prevents RANKL from binding to RANK on osteoclast precursors, inhibiting osteoclast differentiation, function, and survival. The result is suppressed bone turnover, reduced bone resorption markers, and increased bone mineral density. The key benefits are a powerful antiresorptive effect with rapid suppression of osteoclast activity and measurable increases in bone density in conditions where a rapid reduction in bone turnover is desired (e.g., high fracture risk and tumor-related bone loss) [141]. On the other hand, there are potential risks and limitations that need to be considered, such as rebound bone resorption after discontinuation: stopping RANKL inhibition can lead to a rapid increase in osteoclast activity and marked bone loss (rebound phenomenon), which may increase fracture risk unless follow-up therapy is given. There is also a risk of hypocalcemia, particularly in patients with vitamin D deficiency, severe renal impairment, or high bone turnover; calcium and vitamin D status should be optimized before and during therapy [142].

Osteonecrosis of the jaw (ONJ) and atypical femoral fractures are recognized as rare but serious adverse events associated with potent antiresorptives, including RANKL inhibitors [141], in the treatment of bone metastasis of malignant tumors, as reported in pharmacological mechanistic studies and clinical trials. Observational and registry analyses suggest that infection risk may rise when denosumab is used together with other immunomodulatory therapies (e.g., biologic DMARDs), so combined use requires caution. The clinical context should be considered: absolute infection risk depends on patient comorbidities (renal impairment, diabetes, and prior infections), concurrent immunosuppressants, and the treated population (osteoporosis vs. oncology) [143,144].

Despite setbacks, research continues into multi-antigen vaccines targeting *S. aureus*, aiming to prevent deep surgical site infections and recurrent osteomyelitis. Training innate immunity or vaccinating to improve pathogen clearance and reduce recurrence risk includes strategies that prime macrophages/neutrophils or elicit protective adaptive responses [145].

One of the main challenges is ensuring that immunomodulatory therapies do not overly suppress the immune response. A fine balance must be struck between reducing inflammation and preserving the host’s ability to clear the infection. In clinical practice, cytokine-targeted therapies are most likely to be used alongside traditional antimicrobial treatments and surgical debridement. Such differential strategies are critical for managing complex cases, especially those with implant-associated biofilms, where the local immune environment is severely compromised.

Strategies include immune reprogramming to penetrate biofilms, use of matrix-degrading enzymes, and use of delivery systems that enhance immune access to infected niches [146]. For biofilms and intracellular reservoirs, therapies must address biofilm protection and intracellular persistence (e.g., *S. aureus* in osteocytes), which often require host-directed and penetration-enhancing tactics.

Additionally, studies are exploring how biomaterials and immune-modulating therapies can enhance bone regeneration and combat infections. Biomaterials can be engineered to release cytokines, antimicrobial peptides, or immune-polarizing agents. Immunomodulatory biomaterials include scaffolds or coatings that release anti-inflammatory cytokines, immunomodulatory drugs, or antimicrobial peptides while promoting bone regeneration; preclinical work shows reduced infection and improved healing. These scaffolds promote macrophage M2 polarization and reduce inflammatory osteolysis while supporting bone regeneration [147].

Antibacterial polymethylmethacrylate (PMMA) bone cement plays a significant role in the prevention of periprosthetic joint infection (PJI). PMMA cement immobilizes antibacterial comonomers [148].

Another recent study evaluated a different biomaterial, submicron gentamicin sulfate-loaded ultra-high-molecular-weight polyethylene (SM-GS/UHMWPE), as a next-generation antibiotic-eluting spacer for PJI treatment, offering an alternative to antibiotic-loaded bone cement (ALBC) for sustained drug release and reduced systemic toxicity risks [149]. The vitamin E-blended prosthetic biomaterial UHMWPE induced an osteoimmunological response in bone cells that positively affected the osteolysis induced by wear debris, thereby reducing the aseptic loosening of the implants. Vitamin E stabilization of prosthetic biomaterials could contribute to a reduction in oxidation-induced osteolysis and the consequent loosening of prosthetic devices, therefore improving the longevity of total joint replacements [150].

Timing and localization are critical: early modulation may help clearance, while late or systemic immunosuppression can worsen infection. Local delivery (scaffolds and coatings) reduces systemic exposure. The balance between anti-inflammatory and antimicrobial activity is extremely important: suppressing inflammation reduces bone loss but may impair pathogen clearance; combined strategies (immunomodulation + targeted antimicrobials) are preferred. Therapies must address biofilm protection and intracellular persistence (e.g., *S. aureus* in osteocytes), which often require host-directed and penetration-enhancing tactics [136].

Any therapeutic approach must follow the safety and regulatory pathways: novel biomaterials and immune biologics face complex preclinical and clinical testing; combination products (drug + device) have additional regulatory requirements [147].

In conclusion, clinical trials and translational studies continue to evaluate the efficacy and safety of these approaches in bone infections. As our understanding of osteoimmunology deepens, it is hoped that such therapies will move from experimental phases to standard clinical practice.

## Figures and Tables

**Table 1:** Effect of infections on bone resorption and formation, in particular on the cellular pathogenetic mechanism and clinical outcome.

Process	Effect of Infection (Bacterial/Viral)	Cellular Mechanism	Outcome
Bone Resorption	Increased (Osteoclastogenesis)	1. Chronic Inflammation (TNF-alpha, IL-1beta, IL-6) 2. Increase RANKL/OPG Ratio 3. Osteoclast activation/survival	Excessive Bone Loss Bone weakening, Implant Loosening (PJI)
Bone Formation	Decreased/Inhibited	1. Inflammatory Cytokines (TNF-alpha, IL-1beta, IL-6) 2. Inhibit Osteoblast Differentiation/Function	Delayed Healing, Impaired Bone Regeneration, Failure to Repair Defects

**Table 2:** Specific effects of viral and bacterial infections on cellular mechanisms, key pathogenetic components, and clinical consequences.

Infection Type	Key Pathogenic Component	Immune/Cellular Driver	Clinical Consequence
Bacterial (PJI)	Biofilm, LPS (Lipopolysaccharide)	1. Chronic Pro-inflammatory Environment sustains bone loss and prevents healing. 2. Activation of Toll-Like Receptors (TLRs), especially TLR2 and TLR4.	Implant Failure, Irreversible Bone Damage, Therapeutic Challenge.
Viral (HIV)	Chronic Infection, Viral Proteins	1. Persistent Systemic Inflammation (TNF alpha, IL-6). 2. Lymphocyte Dysfunction reduce OPG-expressing B cells 3. Reduce RANKL-expressing B cells.	Osteoporosis and Fracture Risk. Accelerated bone loss.
Viral (COVID-19)	“Cytokine Storm” (Acute/Systemic)	1. Acute release of High IL-6 and TNF-alpha levels 2. Increase RANKL/OPG Ratio linked to bone fragility (correlation with FGF23).	Accelerated Bone Loss, Reduced Bone Mineral Density (BMD), increase Susceptibility to Fractures.

**Table 3:** Immune modulation strategies for preventing and treating bone infections.

Strategy	Primary Mechanism	Main Target	Key Advantage	Mail Limitation
Immunomodulatory biomaterials	Local release of immunoregulatory antimicrobials	Local innate immunity; macrophage polarization;biofilm	Combines infection control and bone repair locally	Complex design; regulatory hurdles
Cytokine modulation	Block or supply cytokines to rebalance inflammation	TNF-alpha, IL-1beta, IL-6, IL-17,RANKL	Direct control of destructive inflammation	Risk of systemic immunosuppression; timing critical
RANKL pathway inhibition	Reduce osteoclastogenesis and bon loss	RANK/RANKL/OPG axis	Protects bone from inflammatory osteolysis	May impair bone remodeling; infection clearance effects uncertain
Trained immunity and vaccines	Enhance innate/adaptive response to pathogens	Neutrophils, macrophages, pathogen specific immune response	Potential long-term pretection against recurrence	Vaccine development for *S. aureus* remains challenging
Host directed biofilm targeting	Reprogramme immune cells to penetrate/disrupt biofilm	Biofilm matrix, phagocyte recruitment	Addresses antibiotic-resistant biofilms	Biofilm heterogeneity; delivery to bone niche difficult

## Data Availability

No new data were created or analyzed in this study. Data sharing is not applicable to this article.
